# Associations of coronary artery calcified plaque density with mortality in type 2 diabetes: the Diabetes Heart Study

**DOI:** 10.1186/s12933-018-0714-z

**Published:** 2018-05-11

**Authors:** Laura M. Raffield, Amanda J. Cox, Michael H. Criqui, Fang-Chi Hsu, James G. Terry, Jianzhao Xu, Barry I. Freedman, J. Jeffrey Carr, Donald W. Bowden

**Affiliations:** 1 Department of Genetics, University of North Carolina at Chapel Hill, 5100 Genetic Medicine Building, 120 Mason Farm Road, Chapel Hill, NC 27599 USA; 2 Center for Diabetes Research, Wake Forest School of Medicine, Winston-Salem, NC USA; 3 Center for Human Genomics, Wake Forest School of Medicine, Winston-Salem, NC USA; 4 Molecular Basis of Disease, Griffith University, Southport, Brisbane, QLD Australia; 5 Department of Family and Preventive Medicine, University of California, San Diego, CA USA; 6 Department of Biostatistical Sciences, Wake Forest School of Medicine, Winston-Salem, NC USA; 7 Department of Radiology and Radiological Sciences, Vanderbilt University, Nashville, TN USA; 8 Department of Internal Medicine—Section on Nephrology, Wake Forest School of Medicine, Winston-Salem, NC USA; 9 Department of Biochemistry, Wake Forest School of Medicine, Winston-Salem, NC USA

**Keywords:** Coronary artery calcification, Plaque density, Type 2 diabetes, Mortality

## Abstract

**Background:**

Coronary artery calcified plaque (CAC) is strongly predictive of cardiovascular disease (CVD) events and mortality, both in general populations and individuals with type 2 diabetes at high risk for CVD. CAC is typically reported as an Agatston score, which is weighted for increased plaque density. However, the role of CAC density in CVD risk prediction, independently and with CAC volume, remains unclear.

**Methods:**

We examined the role of CAC density in individuals with type 2 diabetes from the family-based Diabetes Heart Study and the African American-Diabetes Heart Study. CAC density was calculated as mass divided by volume, and associations with incident all-cause and CVD mortality [median follow-up 10.2 years European Americans (n = 902, n = 286 deceased), 5.2 years African Americans (n = 552, n = 93 deceased)] were examined using Cox proportional hazards models, independently and in models adjusted for CAC volume.

**Results:**

In European Americans, CAC density, like Agatston score and volume, was consistently associated with increased risk of all-cause and CVD mortality (*p* ≤ 0.002) in models adjusted for age, sex, statin use, total cholesterol, HDL, systolic blood pressure, high blood pressure medication use, and current smoking. However, these associations were no longer significant when models were additionally adjusted for CAC volume. CAC density was not significantly associated with mortality, either alone or adjusted for CAC volume, in African Americans.

**Conclusions:**

CAC density is not associated with mortality independent from CAC volume in European Americans and African Americans with type 2 diabetes.

**Electronic supplementary material:**

The online version of this article (10.1186/s12933-018-0714-z) contains supplementary material, which is available to authorized users.

## Background

Many studies have found computed tomography (CT)-based measures of calcified plaque in the coronary arteries (CAC) to be predictive of cardiovascular disease (CVD) events and mortality, independent from traditional CVD risk factors [1–5]. CAC is also a powerful independent risk factor for CVD and mortality in individuals with type 2 diabetes (T2D) [6–9], with elevated CAC burden in diabetic patients compared to non-diabetic individuals [10]. This is of particular interest due to the elevated CVD risk in individuals with T2D, with mortality risk from CVD increased two- to fourfold and approximately 68% of T2D-affected individuals age 65 or older succumbing to CVD [11].

Recent work from the Multi-Ethnic Study of Atherosclerosis (MESA) found that increased CAC density, in models adjusted for plaque volume, was associated with a decreased risk of coronary heart disease (CHD) events (HR of 0.73, 95% CI 0.58–0.91 per standard deviation increase) and all CVD events, with improved risk prediction for CHD and CVD events with the inclusion of density [12]. These results were contrary to the assumptions of using Agatston scores to assess CAC, as those scores are weighted for increased density [13, 14], and the results have been confirmed in re-analysis of the MESA dataset with additional events from 11 years median follow-up [15]. Further study of the role of calcified plaque density in CVD risk appears to be warranted. We evaluated the associations of CAC density measures with incident mortality in a high risk population with T2D, including participants of both European American (EA) and African American (AA) descent from the Diabetes Heart Study (DHS) and African American-Diabetes Heart Study (AA-DHS).

## Methods

### Study design and sample

The DHS recruited T2D-affected siblings without advanced renal insufficiency, as well as their unaffected siblings when possible, from outpatient internal medicine and endocrinology clinics at Wake Forest Baptist Medical Center and from the community from 1998 through 2005; this initial DHS cohort included 1221 self-reported EA individuals and 222 self-reported AA participants. Differences in the distribution of vascular calcification between EA and AA participants, with AA participants generally having lower vascular calcification burden despite higher levels of traditional CVD risk factors [16], an observation supported by many additional studies [17–19], prompted the development of the independent AA-DHS Study. AA-DHS recruited additional unrelated African American participants with T2D from 2007 to 2010. Recruitment criteria and objectives of the DHS family of studies have been reported [20]. T2D was defined in DHS studies as diabetes developing after the age of 35 years (or after the age of 30 in AA participants) initially treated with changes in diet and exercise and/or oral agents, in the absence of historical evidence of ketoacidosis or initial treatment with insulin. Individuals with prior evidence of CVD were included. Fasting glucose and glycated hemoglobin (HbA_1C_) concentrations were assessed at the exam visit.

Participant examinations for the DHS and AA-DHS included interviews for medical history and health behaviors, anthropometric measures, resting blood pressure, electrocardiography, fasting blood sampling for laboratory analyses, and spot urine collection. As described, CAC was assessed using CT, summing the left main, left anterior descending, circumflex, posterior descending, and right coronary arteries [21–23]. CT scans were performed on multi-detector CT scanners with cardiac gating in chest scans, with a CT slice thickness of 2.5 mm for AA-DHS and 2.5 or 3 mm, depending on the scanner used, for DHS. The protocol for CAC imaging was the same as in two large population-based studies of subclinical cardiovascular disease, MESA and the Coronary Artery Risk Development in Young Adults (CARDIA) Study [24–26]; however, the DHS used the FDA approved and validated GE Healthcare Smartscore software program, improving assessment of mass scores.

Mortality was assessed using the National Social Security Death Index. When possible, copies of death certificates were obtained from county or state Vital Records Offices to determine cause of death. Cause of death was categorized based on death certificates as CVD mortality [myocardial infarction (MI), congestive heart failure, cardiac arrhythmia, sudden cardiac death, peripheral vascular disease, and stroke] or as mortality from other causes. However, for 15 EA participants and 8 AA participants, information on cause of death could not be obtained; these 23 participants were excluded from analyses of CVD mortality. For deceased participants, length of follow-up was determined from the date of initial study visit to date of death. For all other participants the length of follow-up was determined from the date of the initial study visit to December 31, 2013.

For our main analysis, we included only individuals affected by T2D with non-missing covariate data and non-zero values for the relevant CAC values at the 90 Hounsfield unit (HU) threshold, which is more sensitive but less specific than the frequently used 130 HU threshold. This included a total of 902 EA participants from 448 families from the DHS and 552 AA participants from 483 families from the DHS and AA-DHS, including 139 individuals from 70 families from the DHS and 413 unrelated individuals from the AA-DHS. A supplemental analysis of 816 EA participants and 446 AA participants with non-zero Agatston scores at the 130 HU threshold was also performed.

### Statistical analysis

Associations were examined for five measures of calcified plaque burden, including volume, Agatston score at the 90 HU and 130 HU thresholds, and two density measures. The density measure used in all main analyses was calculated by dividing mass over volume. As a supplemental analysis, an alternate density measure calculated as Agatston score over plaque area (calculated as volume divided by CT slice thickness, which was either 2.5 or 3 mm depending on the scanner used) was analyzed. This alternate density measure is similar to the measure analyzed in MESA, which did not have access to reliable mass scores [12]. Some plaque measures were transformed prior to analysis. In EA and AA participants, volume and Agatston scores were ln transformed and the alternate density measure calculated as Agatston divided by area was squared to better approximate a normal distribution (Additional file 1: Figure S1). Untransformed summary statistics are presented in Table 1. Non-parametric Spearman correlation coefficients were calculated to determine relationships between these calcified plaque measures.

**Table 1:** Demographic characteristics of European American and African American participants with type 2 diabetes from the Diabetes Heart Study and African American Diabetes-Heart Study cohorts with non-zero coronary artery calcification burden and complete covariate data for analysis models

Trait	European Americans (n = 902)			African Americans (n = 552)		
	Mean (SD) or %	Median (range)	n	Mean (SD) or %	Median (range)	N
Age (years)	62.8 (9)	63.3 (34.2, 86)	902	57.4 (9.3)	57.5 (35, 86)	552
Female sex (%)	50.4		902	59.8		552
Current smoking (%)	16.5		902	23.9		552
Past smoking (%)	43.6		902	36.8		552
History of myocardial infarction (%)	22.4		894	11.9		547
History of cardiovascular disease (%)	44.7		894	35.7		526
Incident all-cause mortality (%)	31.7		902	16.9		552
Incident cardiovascular disease mortality (%)	14.9		887	7.7		544
Follow-up time (years)	9.5 (3.1)	10.2 (0.3, 15.6)	902	6 (3)	5.2 (0.3, 16)	552
Body mass index (kg/m^2^)	32.3 (6.5)	31.3 (17.1, 58)	902	35.3 (8.2)	34 (17.1, 77.5)	552
Glucose (mg/dL)	148.2 (56.2)	135.5 (16, 463)	900	149.6 (65.3)	134 (32, 524)	552
Glycated hemoglobin (%)	7.5 (1.6)	7.2 (4.6, 16.6)	896	8.2 (2.1)	7.7 (4.4, 21.8)	543
Glycated hemoglobin (mmol/mol)	59 (17.7)	55.2 (26.8, 157.9)	896	66.2 (23.2)	60.7 (24.6, 214.8)	543
Diabetes duration (years)	10.6 (7.2)	8 (0, 46)	888	10.7 (7.9)	8.5 (0, 52)	552
CAC mass score (mg, 90 HU)	1960 (3414)	562 (0.5, 50,415)	889	1028 (2025)	161 (1, 15,527)	552
CAC volume score (mm^3^, 90 HU)	899 (1328)	361 (1, 15,569)	902	432 (784)	90 (1, 6103)	552
CAC Agatston score (90 HU)	1031 (1640)	365 (0.5, 20,563)	901	497 (956)	82 (0.5, 7502)	551
CAC Agatston score (130 HU)	828 (1277)	331 (0.5, 16,287)	816	446 (772)	110 (0.5, 5693)	446
CAC density (mg/mm^3^, 90 HU)	1.71 (0.76)	1.74 (0.02, 4.46)	889	1.8 (0.65)	1.75 (0.9, 5.39)	552
Alternate CAC density (Agatston score/area, Hounsfield unit category units, 90 HU)	2.25 (0.83)	2.51 (0.63, 4.29)	901	2.09 (0.87)	2.35 (0.5, 3.42)	551
Total cholesterol (mg/dL)	184.5 (43.1)	180 (65, 391)	902	183.5 (45.6)	178 (81, 428)	552
HDL (mg/dL)	42.3 (12)	41 (8, 98)	902	48.3 (14)	46 (18, 115)	552
Systolic blood pressure (mmHg)	140 (19.1)	138.5 (94, 260)	902	136.7 (19.4)	134.3 (85, 211)	552
Diastolic blood pressure (mmHg)	72.3 (10.2)	71.5 (36.5, 106)	902	77.3 (11.6)	77 (48.5, 122)	552
High blood pressure medications (%)	76.4		902	84.1		552
Statin use (%)	43.8		902	49.5		552
Oral hypoglycemic medications (%)	79.5		902	76.3		552
Insulin use (%)	27.5		902	42.2		552

*CAC* coronary artery calcified plaque, *HU* Hounsfield unit

Associations with incident all-cause and CVD mortality were assessed using Cox proportional hazards models with sandwich-based variance estimation, due to the family structure of the DHS cohort. Models were adjusted for age, sex, study (DHS and AA-DHS, for AA participants only), statin use, ln transformed total cholesterol, square root transformed high-density lipoprotein cholesterol (HDL), systolic blood pressure (SBP), high blood pressure medication use, and current smoking. A minimally adjusted model (adjusted for age, sex, and, in African Americans, study only) was also assessed; results were similar and are not shown. As a supplemental analysis, self-reported prior CVD events were examined using marginal models with generalized estimating equations with a sandwich estimator of the variance under exchangeable correlation. For self-reported prior CVD, we analyzed (a) self-reported history of MI, and (b) a composite measure including MI, angina, or stroke, history of vascular procedures including coronary angioplasty, coronary artery bypass graft, or endarterectomy, or Q wave abnormalities indicative of prior MI using the Minnesota code. All analyses were performed in SAS 9.3.

## Results

Demographic characteristics of AA and EA participants in the DHS and AA-DHS included in calcified plaque density analyses are summarized in Table 1. Mean diabetes duration was 10.6 ± 7.2 years [mean ± standard deviation (SD)] in EA participants and 10.7 ± 7.9 years in AA participants. 44.7% of EA participants and 35.7% of AA participants had a history of CVD, and mean BMI was > 30 kg/m^2^ in both groups. While burden of subclinical CVD was extensive in both groups, EA participants had higher median calcified plaque volume (361) than AA participants (90), consistent with prior reports [16–18]. Using a 90 HU threshold, there were 901 EA participants with a non-zero Agatston score (median 365) and 551 AA participants with a non-zero score (median 82); as expected, fewer individuals had a non-zero Agatston score at the less sensitive but more specific 130 HU threshold, with 816 EA participants with a non-zero Agatston score (median 331) and 446 AA participants with a non-zero score (median 110). Incident all-cause mortality was greater for EA participants (31.7%) than AA participants (16.9%) as well, likely in part due to longer follow-up time in EA participants.

In both EA (Additional file 1: Table S1) and AA (Additional file 1: Table S2) participants, the CAC measures are highly correlated, with volume and Agatston score at the 90 HU and 130 HU threshold very highly correlated in both groups (r > 0.95). Volume and Agatston score measures were also significantly correlated with density in AA and in EA participants (r > 0.66).

In EA participants (Table 2), all CAC measures, including volume, Agatston scores at both the 90 HU and 130 HU thresholds, and density, were associated with increased risk of all-cause and CVD mortality (*p* ≤ 0.002) in models adjusted for age, sex, statin use, total cholesterol, HDL, SBP, high blood pressure medication use, and current smoking. When density and volume measures were included in the same model (Table 3), plaque volume remained consistently associated with elevated mortality (*p* ≤ 3.70 × 10^−4^), but associations with plaque density were attenuated to non-significance (*p* ≥ 0.252). In the smaller AA sample, plaque volume and Agatston scores were associated with elevated risk of all-cause and CVD mortality (*p* ≤ 0.046), but plaque density was not significantly associated with mortality risk (*p* ≥ 0.106) (Table 2). Similar to EAs, in models including both density and volume measures, volume was associated with elevated mortality risk in AAs (*p* ≤ 0.003), but no significant association with density was observed (*p* ≥ 0.117) (Table 3).

**Table 2:** Associations with incident all-cause and cardiovascular disease (CVD) mortality for coronary artery calcification measures analyzed in independent models in European American and African American participants with type 2 diabetes

Outcome	CAC measure	European Americans					African Americans				
		Hazard ratio	95% confidence interval		p-value	n	Hazard ratio	95% confidence interval		p-value	n
All-cause mortality	Volume (90 HU)	1.67	1.37	2.03	3.08 × 10^−7^	902	1.48	1.14	1.92	0.003	552
All-cause mortality	Agatston score (90 HU)	1.65	1.35	2.02	1.02 × 10^−6^	901	1.45	1.12	1.88	0.005	551
All-cause mortality	Agatston score (130 HU)	1.54	1.27	1.87	1.42 × 10^−5^	816	1.27	1.01	1.61	0.046	446
All-cause mortality	Density (90 HU)	1.31	1.12	1.53	6.98 × 10^−4^	889	1.13	0.92	1.38	0.233	552
CVD mortality	Volume (90 HU)	1.93	1.45	2.57	6.58 × 10^−6^	887	2.00	1.25	3.21	0.004	544
CVD mortality	Agatston score (90 HU)	1.86	1.39	2.49	3.46 × 10^−5^	886	1.98	1.25	3.13	0.004	543
CVD mortality	Agatston score (130 HU)	1.68	1.25	2.27	5.74 × 10^−4^	801	1.84	1.20	2.84	0.006	438
CVD mortality	Density (90 HU)	1.39	1.13	1.70	0.002	875	1.26	0.95	1.66	0.106	544

Hazard ratios for mortality associations reported per standard deviation change in coronary artery calcification measures. Models adjusted for age, sex, statin use, total cholesterol, HDL, systolic blood pressure, high blood pressure medication use, and current smoking; analyses additionally adjusted for study (Diabetes Heart Study or African American Diabetes Heart Study) in African Americans

*HU* Hounsfield unit

**Table 3:** Associations with incident all-cause and cardiovascular disease (CVD) mortality for density and volume measures analyzed in the same model in European American and African American participants with type 2 diabetes

Outcome	CAC measure	European Americans					African Americans				
		Hazard ratio	95% confidence interval		p-value	n	Hazard ratio	95% confidence interval		p-value	n
All-cause mortality	Volume	1.60	1.29	1.99	2.25 × 10^−5^	889	1.76	1.22	2.53	0.003	552
	Density	1.10	0.94	1.28	0.225	889	0.77	0.55	1.07	0.117	552
CVD mortality	Volume	1.78	1.30	2.44	3.70 × 10^−4^	875	2.56	1.40	4.67	0.002	544
	Density	1.13	0.92	1.39	0.252	875	0.69	0.43	1.10	0.121	544

Hazard ratios for mortality associations reported per standard deviation change in coronary artery calcification measures. Models adjusted for age, sex, statin use, total cholesterol, HDL, systolic blood pressure, high blood pressure medication use, and current smoking; analyses additionally adjusted for study (Diabetes Heart Study or African American Diabetes Heart Study) in African Americans. All calcification measures were derived using a 90 HU (Hounsfield unit) threshold

As a supplementary analysis, we also analyzed associations of CAC measures with self-reported history of CVD and MI. In EA participants, all CAC measures, including density, were associated with higher odds of having a history of CVD and MI events (*p* ≤ 4.65 × 10^−6^), with similar results in AA participants (*p* ≤ 1.84 × 10^−4^) (Additional file 1: Table S3). With density and volume measures included in the same model, in EA participants both volume and density were associated with higher odds of prior CVD and MI (*p* ≤ 0.046); trends were similar but density was not significantly associated in AA participants (Additional file 1: Table S4).

Finally, to increase comparability of our results with the prior analysis of calcified plaque density in the MESA cohort [12], we repeated our analyses with an alternate measure of plaque density (Agatston score over area), which was derived in MESA as reliable mass measurements were unavailable. Results were broadly similar; higher density was associated with higher CVD mortality and higher odds of history of CVD and MI in EA and AA participants (*p* ≤ 0.014), as well as with higher all-cause mortality in EAs (*p* = 9.62 × 10^−6^) (Additional file 1: Table S5). Adjusting for volume, no significant associations with this alternate density measure remained (Additional file 1: Table S6).

## Discussion

The utility of CAC, usually assessed using Agatston or volume measures, in predicting CVD events and mortality is well-established in the general population [1–5] and in those with T2D [6–9]. Questions remain concerning whether CAC plaque density adds to the predictive power of CAC volume. This analysis from the DHS in both EA and AA participants with T2D assessed associations of multiple CAC measures, including plaque volume, Agatston scores, and density, with incident mortality. We found that all of these CAC measures, including density, were associated higher risk of all-cause and CVD mortality in EA participants. In models adjusted for volume, however, no independent associations with density measures remained. Results were broadly similar in AA participants, though no association of density unadjusted for volume with mortality risk was observed. These results do not suggest a consistent association of CAC density with mortality independent of volume in patients with T2D.

While our results do not suggest an independent relationship of calcified plaque density with mortality, further study is needed to assess how the characteristics of calcified and non-calcified plaque may contribute to CVD risk. Our scans are done with non-contrast CT and we can comment on characteristics such as density for calcified plaque only, not the non-calcified portions; total coronary plaque area is generally ~ 5 times greater than calcified plaque area [27]. We did not assess more specific plaque characteristics such as the fibrous cap thickness or consider differential mortality risk based on characteristics such as lesion number and location in the coronary arteries [28]. Further understanding of plaque characteristics in multiple patient populations, including those with diabetes, may also help evaluate the usefulness of alternate calcified plaque scoring methods and density calculations [29–32], which may have advantages over the Agatston method in certain clinical and research settings (such as increased speed in CAC assessment [32, 33], or increased sensitivity for detecting very small plaques [29]). Plaque characteristics, as opposed to simply total plaque, may also modify observed associations with CVD events. In individuals with diabetes [34], as well as other patient populations [35, 36], some reports suggest that very high CAC scores, which may represent larger, denser plaques, are associated with increased risk of more stable CVD phenotypes (such as angina) versus sudden death or MI; however, in our population, previous work has shown very high mortality rates in individuals with T2D and CAC > 1000 [37], suggesting substantial CVD burden in this population. Further study is needed to determine what levels of CAC and CAC characteristics are associated with particular CVD events, particularly in individuals with T2D.

Results in the DHS differ from those previously observed in MESA, in which the largest existing multi-ethnic study of CAC plaque density was performed. Calcified plaque density unadjusted for volume was not predictive of CHD or CVD events in MESA, but, when models included both CAC volume and CAC density, increased plaque density was associated with a decreased risk of CHD and CVD events [12]. In the DHS, calcified plaque density unadjusted for volume was predictive of higher mortality risk (in EA participants); however, in models adjusted for volume, plaque density was not associated with mortality. There was a non-significant trend towards reduced risk of all-cause and CVD mortality with increased density in models adjusted for volume in African Americans, which would be concordant with the MESA results.

A number of differences exist between our analysis of the DHS and AA-DHS studies and the analysis in MESA. Most notably, our study was limited to participants with T2D, while MESA included only 17.9% T2D-affected participants. However, there was no significant interaction for diabetes in the most recent MESA analysis of density adjusted for volume [15]. The DHS is characterized by a high average burden of CAC, with a mean CAC Agatston score at the 130 HU threshold of 828 in EA participants and 446 in AA participants, compared to a mean CAC Agatston score of 293 for MESA participants, which recruited individuals free of clinical CVD at baseline. MESA had relatively low rates of statin use in those with non-zero CAC (20.1% of participants), likely due to lack of clinical CVD at baseline, while many EA and AA DHS participants reported a history of clinical CVD at baseline (> 35% in both groups), and statin use was higher (> 40%). Statin use may impact the differing relationships of CAC density and CVD in DHS and MESA, though both analyses did adjust for statin use; some studies indicate statins may increase CAC progression [38] and may impact CAC density, for example by reducing low attenuation plaque volume [39, 40]. A recent analysis from MESA suggested that statin use may attenuate the association between calcified plaque density and incident CVD in those with diabetes or metabolic syndrome [41]. We used a more intuitive mass over volume measure for density for our main analyses, though results were similar with an alternate density measure calculated as Agatston score over area (similar to that analyzed in MESA) in supplementary analyses. The Agatston score weighting of area is not based on a true measured density of each pixel, but instead incorporates a weighted measure from 1 to 4 based on the highest density pixel, limiting the interpretability of the Agatston/area estimate [13]. Other differences include smaller sample size, stratification by self-reported ethnicity (supported by significant interactions between self-reported ancestry and calcified plaque measures (*p* < 0.05) in joint models), higher correlation between density and volume (though no extreme collinearity problems (variance inflation factor > 4) were observed), and no data for incident events. We also acknowledge that inaccuracies in cause of death data from death certificates have been documented [42, 43]; under- or over-reporting by physicians may be a concern for our CVD mortality measure. It should be noted, however, that this ambiguity is not present when assessing all-cause mortality.

Analyses in MESA used the less sensitive but slightly more specific [30] 130 HU threshold for CAC assessment, as compared to use of a 90 HU threshold in the DHS cohorts, which may have contributed to different results between reports, as inclusion of more edge voxels at the 90 HU threshold can impact the measured area, volume, and density. As Agatston scores, but not volume and mass, were recorded in the DHS cohorts at both the 130 and 90 HU thresholds, we attempted to address this difference by limiting our analysis of density, Agatston score, and volume measures from the 90 HU threshold to only those with a non-zero Agatston score at the 130 HU threshold (n = 816 for EA participants, n = 446 for AA participants). Results were similar to the analysis in the full sample, with results displayed in Additional file 1: Tables S7 and S8. We ran similar models in European participants with all participants with an Agatston score less than 10 at the 130 HU threshold excluded. Again, results were similar, suggesting that small, potentially low density calcified plaques are not overly influencing our results.

## Conclusions

In the T2D-affected DHS populations higher CAC plaque density was not consistently associated with risk of incident mortality in models adjusted for plaque volume. These results suggest that the role of CAC density may differ in the high CVD risk population of individuals affected by T2D; further study is needed to determine whether CAC density is independently predictive of CVD risk and its direction of effect in different patient populations. Longitudinal analyses of changes in CAC plaque density remain an important future goal.

## Additional file

**Additional file 1.** Additional figure and tables.

## Abbreviations

AA: African American
AA-DHS: African American-Diabetes Heart Study
CVD: cardiovascular disease
CT: computed tomography
CAC: coronary artery calcified plaque
CHD: coronary heart disease
DHS: Diabetes Heart Study
EA: European American
HDL: high-density lipoprotein cholesterol
HU: Hounsfield unit
MESA: Multi-Ethnic Study of Atherosclerosis
MI: myocardial infarction
SD: standard deviation
SBP: systolic blood pressure
T2D: type 2 diabetes
