# Supplementary material for: Associations of coronary artery calcified plaque density with mortality in type 2 diabetes: the Diabetes Heart Study
Source: Cardiovasc Diabetol. 2018 May 11;17:67. doi: 10.1186/s12933-018-0714-z (PMC5946410; doi:10.1186/s12933-018-0714-z)
Supplement: Supplementary file 1 — Additional file 1. Additional figure and tables. [file 12933_2018_714_MOESM1_ESM.docx]

Figure S1: Histograms of plaque measures (volume, Agatston score, density, alternate density (Agatston/area)), in European (a.) and African Americans (b.) before and after transformations to approximate normality.


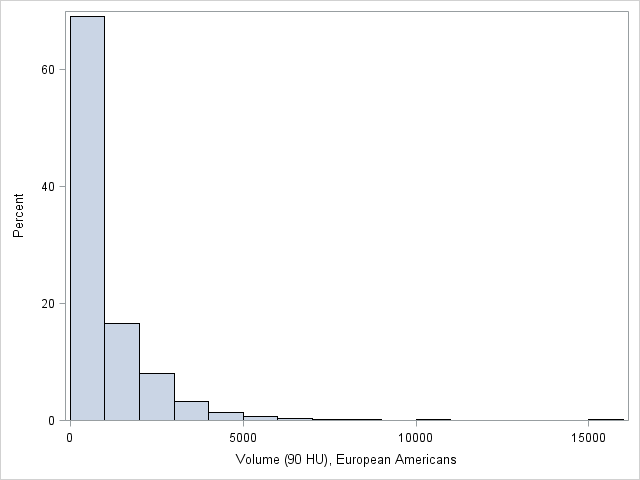


a.1. Volume (90 HU), Shapiro-Wilk statistic 0.657, p<0.0001


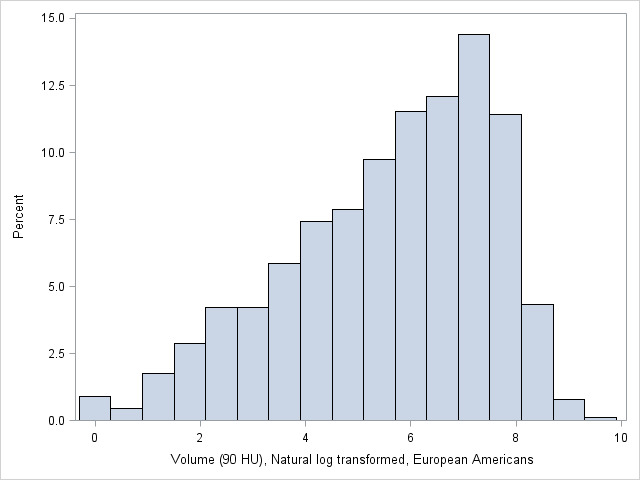


a.2. Volume (90 HU), Natural log transformed, Shapiro-Wilk statistic 0.956, p<0.0001


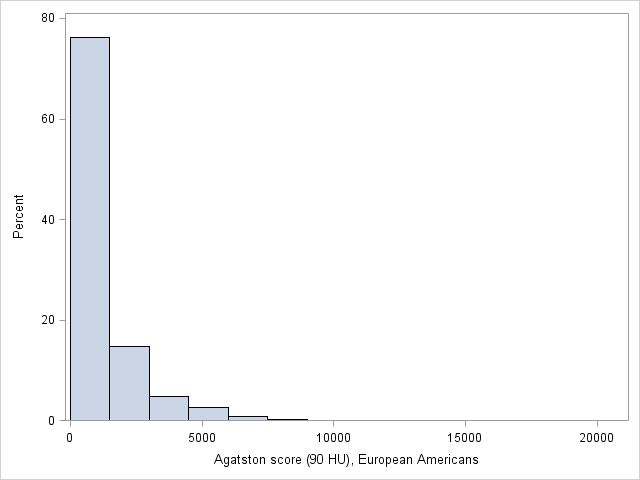


a.3. Agatston score (90 HU), Shapiro-Wilk statistic 0.629, p<0.0001


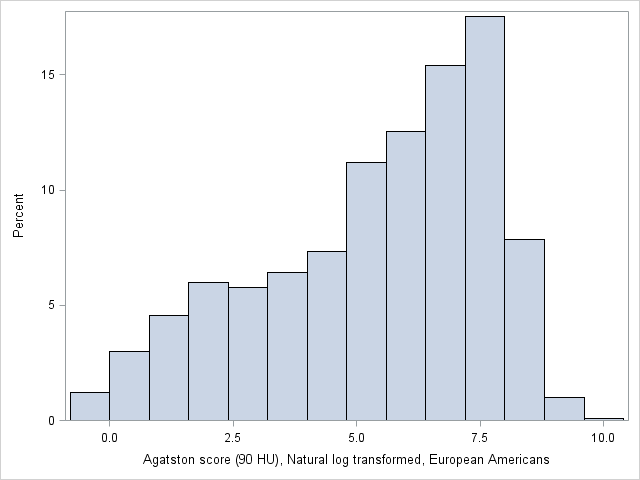


a.4. Agatston score (90 HU), Natural log transformed, Shapiro-Wilk statistic 0.941, p<0.0001


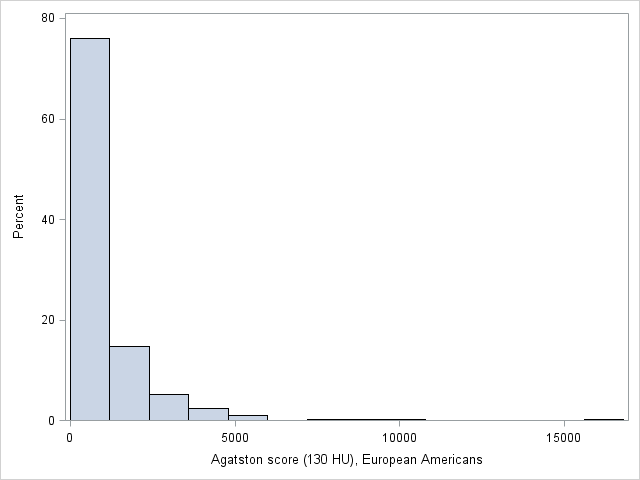


a.5. Agatston score (130 HU), Shapiro-Wilk statistic 0.636, p<0.0001


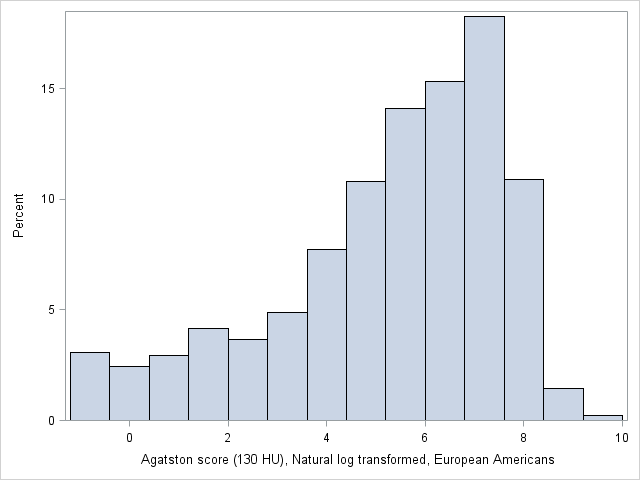


a.6. Agatston score (130 HU), Natural log transformed, Shapiro-Wilk statistic 0.920, p<0.0001


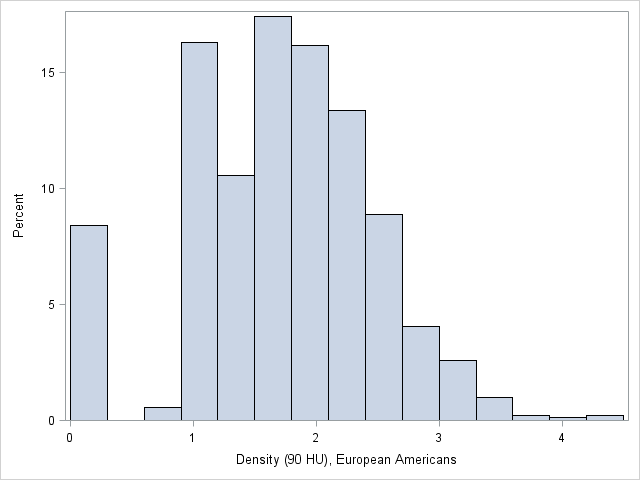


a.7. Density (90 HU), Shapiro-Wilk statistic 0.978, p<0.0001


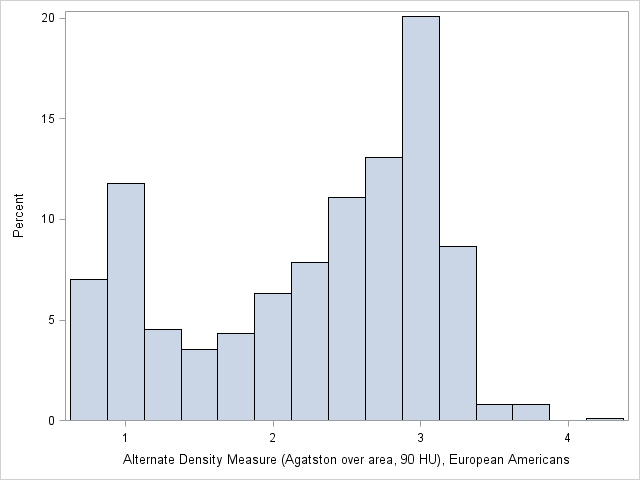


a.8. Alternate Density Measure (Agatston over area, 90 HU), Shapiro-Wilk statistic 0.903, p<0.0001


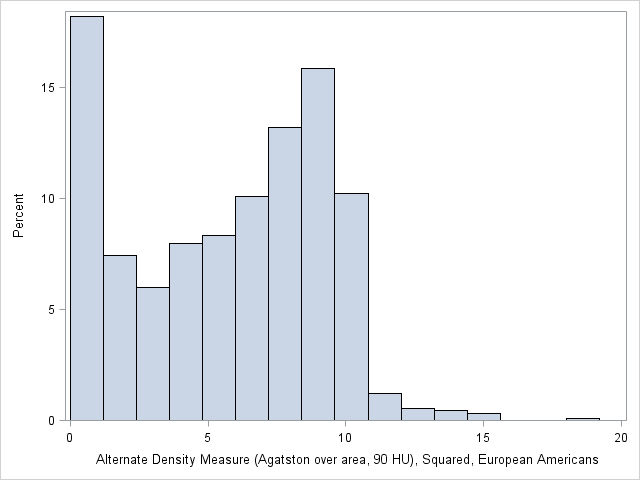


a.9. Alternate Density Measure (Agatston over area, 90 HU), Squared, Shapiro-Wilk statistic 0.931, p<0.0001


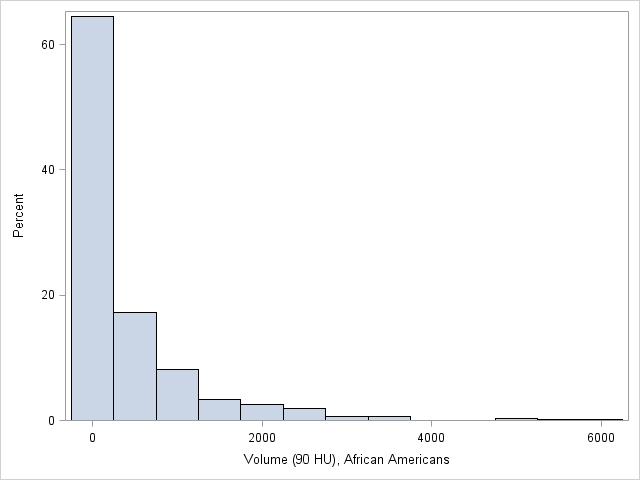


b.1. Volume (90 HU), Shapiro-Wilk statistic 0.591, p<0.0001


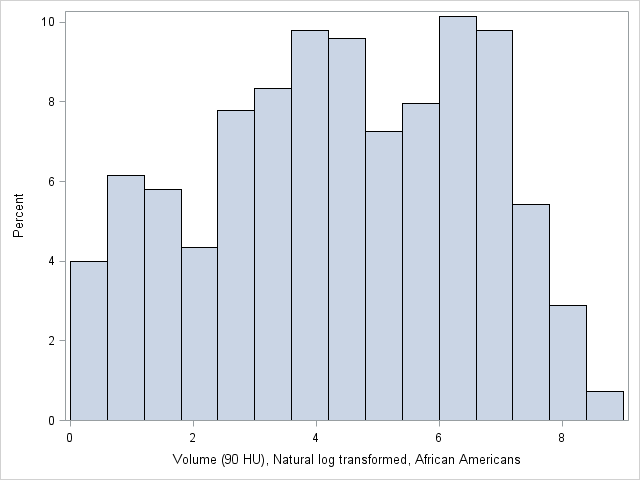


b.2. Volume (90 HU), Natural log transformed, Shapiro-Wilk statistic 0.972, p<0.0001


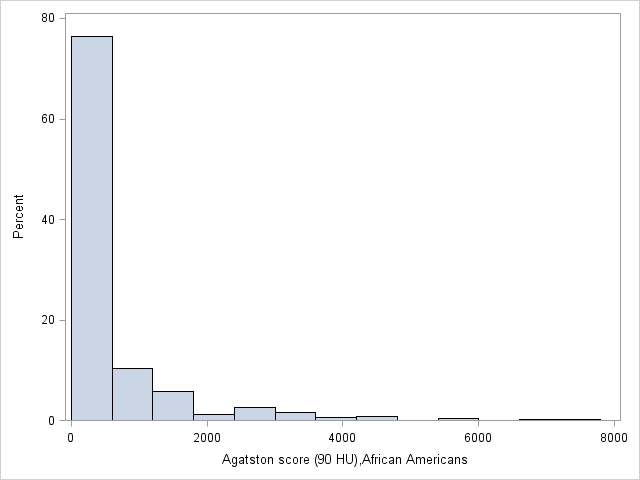


b.3. Agatston score (90 HU), Shapiro-Wilk statistic 0.570, p<0.0001


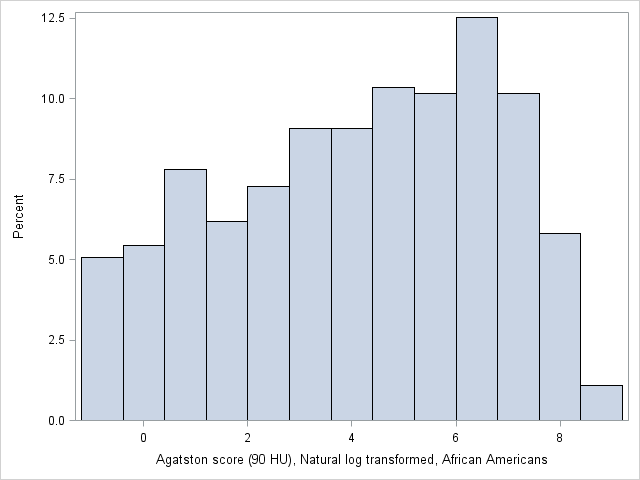


b.4. Agatston score (90 HU), Natural log transformed, Shapiro-Wilk statistic 0.956, p<0.0001


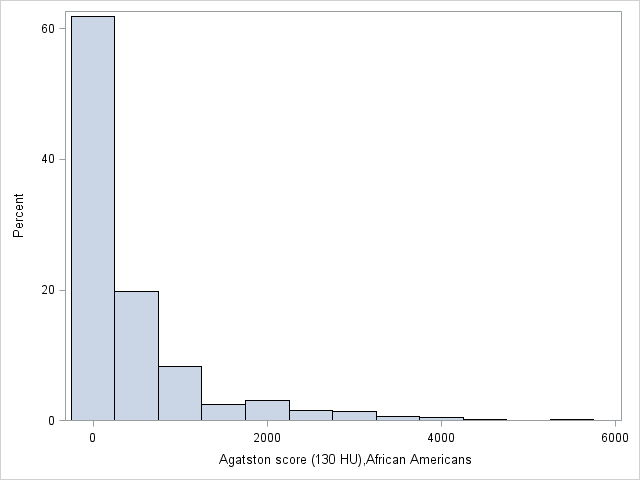


b.5. Agatston score (130 HU), Shapiro-Wilk statistic 0.616, p<0.0001


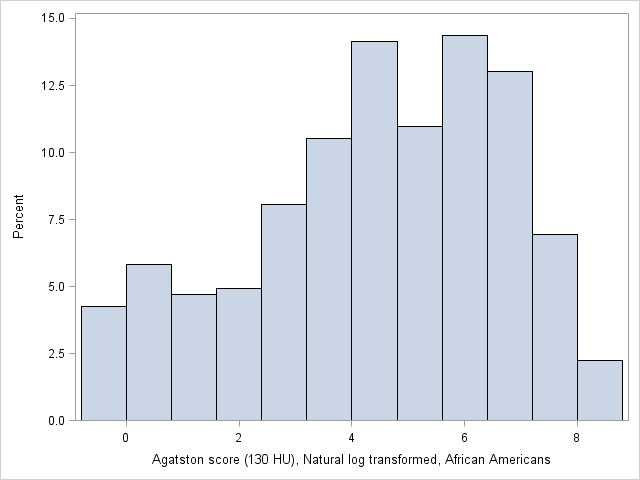


b.6. Agatston score (130 HU), Natural log transformed, Shapiro-Wilk statistic 0.959, p<0.0001


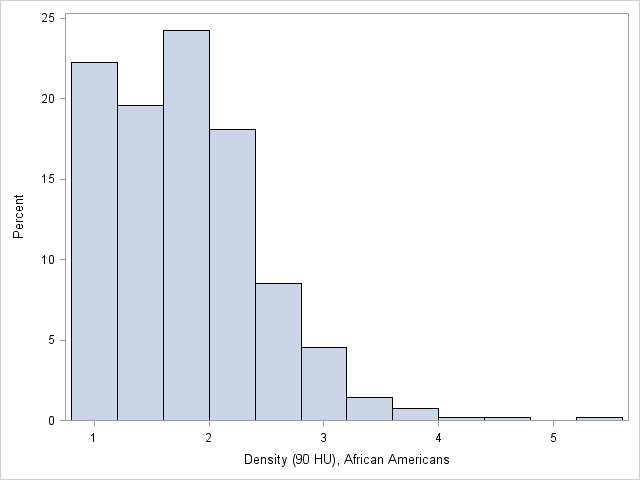


b.7. Density (90 HU), Shapiro-Wilk statistic 0.927, p<0.0001


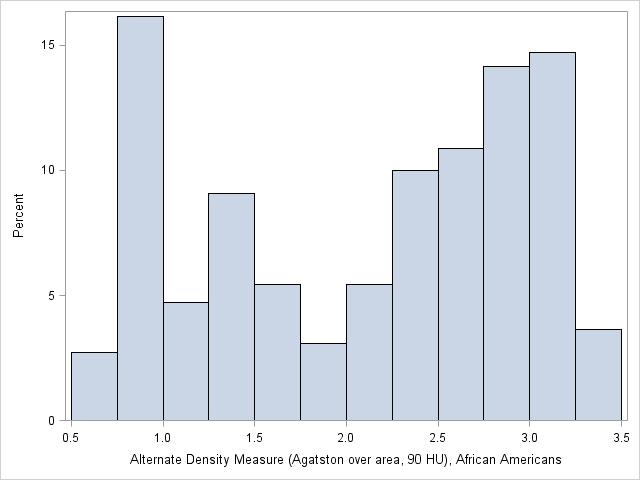


b.8. Alternate Density Measure (Agatston over area, 90 HU), Shapiro-Wilk statistic 0.899, p<0.0001


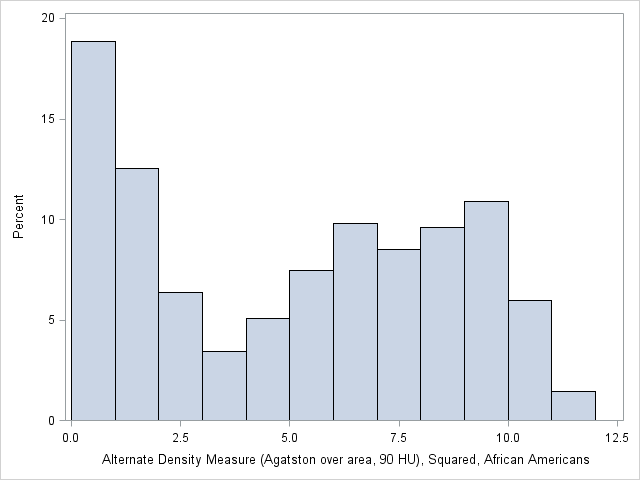


b.9. Alternate Density Measure (Agatston over area, 90 HU), Squared, Shapiro-Wilk statistic 0.907, p<0.0001

Table S1: Spearman correlation coefficients for transformed non-zero values of coronary artery calcification measures in European American participants with type 2 diabetes. HU, Hounsfield unit.

|  | **Volume (90 HU)** | **Agatston score (90 HU)** | **Agatston score (130 HU)** | **Density (90 HU)** | **Alternate Density Measure (Agatston over area, 90 HU)** |
| --- | --- | --- | --- | --- | --- |
| **Volume (90 HU)** | 1.000 | 0.996 | 0.986 | 0.664 | 0.815 |
| **Agatston score (90 HU)** |  | 1.000 | 0.996 | 0.690 | 0.855 |
| **Agatston score (130 HU)** |  |  | 1.000 | 0.678 | 0.836 |
| **Density (90 HU)** |  |  |  | 1.000 | 0.734 |
| **Alternate Density Measure (Agatston over area, 90 HU)** |  |  |  |  | 1.000 |

*All correlations significant (p <0.0001)

Table S2: Spearman correlation coefficients for transformed non-zero values of coronary artery calcification measures in African American participants with type 2 diabetes.

|  | **Volume (90 HU)** | **Agatston score (90 HU)** | **Agatston score (130 HU)** | **Density (90 HU)** | **Alternate Density Measure (Agatston over area, 90 HU)** |
| --- | --- | --- | --- | --- | --- |
| **Volume (90 HU)** | 1.000 | 0.993 | 0.952 | 0.713 | 0.793 |
| **Agatston score (90 HU)** |  | 1.000 | 0.981 | 0.759 | 0.851 |
| **Agatston score (130 HU)** |  |  | 1.000 | 0.705 | 0.829 |
| **Density (90 HU)** |  |  |  | 1.000 | 0.885 |
| **Alternate Density Measure (Agatston over area, 90 HU)** |  |  |  |  | 1.000 |

*All correlations significant (p <0.0001)

Table S3: Associations with history of CVD and myocardial infarction (MI) for coronary artery calcification measures analyzed in independent models in European American and African American participants with type 2 diabetes. Odds ratios for CVD and MI history reported per standard deviation change in coronary artery calcification measures. Models adjusted for age, sex, statin use, total cholesterol, HDL, systolic blood pressure, high blood pressure medication use, and current smoking; analyses additionally adjusted for study (Diabetes Heart Study or African American Diabetes Heart Study) in African Americans. HU, Hounsfield unit.

|  |  | **European Americans** | | | | | **African Americans** | | | | |
| --- | --- | --- | --- | --- | --- | --- | --- | --- | --- | --- | --- |
| **Outcome** | **CAC Measure** | **Odds Ratio** | **95% Confidence Interval** | | **P-value** | **n** | **Odds Ratio** | **95% Confidence Interval** | | **P-value** | **n** |
| **History of CVD** | **Volume (90 HU)** | 2.20 | 1.78 | 2.72 | 4.49 x 10^-13^ | 894 | 1.98 | 1.54 | 2.53 | 6.66 x 10^-8^ | 526 |
| **History of CVD** | **Agatston score (90 HU)** | 2.16 | 1.75 | 2.66 | 9.33 x 10^-13^ | 893 | 1.96 | 1.54 | 2.50 | 6.66 x 10^-8^ | 525 |
| **History of CVD** | **Agatston score (130 HU)** | 2.14 | 1.68 | 2.71 | 3.39 x 10^-10^ | 808 | 1.82 | 1.40 | 2.37 | 7.46 x 10^-6^ | 429 |
| **History of CVD** | **Density (90 HU)** | 1.65 | 1.39 | 1.94 | 5.22 x 10^-9^ | 881 | 1.87 | 1.45 | 2.41 | 1.51 x 10^-6^ | 526 |
| **History of MI** | **Volume (90 HU)** | 3.57 | 2.51 | 5.09 | 1.92 x 10^-12^ | 894 | 4.02 | 2.24 | 7.22 | 3.16 x 10^-6^ | 547 |
| **History of MI** | **Agatston score (90 HU)** | 3.49 | 2.42 | 5.05 | 2.93 x 10^-11^ | 893 | 4.07 | 2.19 | 7.57 | 9.42 x 10^-6^ | 546 |
| **History of MI*** | **Agatston score (130 HU)*** | 3.59 | 2.41 | 5.35 | 3.61 x 10^-10^ | 809 | 3.57 | 1.83 | 6.97 | 1.84 x 10^-4^ | 443 |
| **History of MI** | **Density (90 HU)** | 1.76 | 1.38 | 2.24 | 4.65 x 10^-6^ | 881 | 2.21 | 1.63 | 2.99 | 2.90 x 10^-7^ | 547 |

*Model assumed independent, not exchangeable, correlation structure, in African Americans.

Table S4: Associations with history of CVD and myocardial infarction (MI) for density and volume measures analyzed in the same model in European American and African American participants with type 2 diabetes. Odds ratios for CVD and MI history reported per standard deviation change in coronary artery calcification measures. Models adjusted for age, sex, statin use, total cholesterol, HDL, systolic blood pressure, high blood pressure medication use, and current smoking; analyses additionally adjusted for study (Diabetes Heart Study or African American Diabetes Heart Study) in African Americans. All calcification measures were derived using a 90 HU (Hounsfield unit) threshold.

|  |  | **European Americans** | | | | | **African Americans** | | | | |
| --- | --- | --- | --- | --- | --- | --- | --- | --- | --- | --- | --- |
| **Outcome** | **CAC measure** | **Odds Ratio** | **95% Confidence Interval** | | **P-value** | **n** | **Odds Ratio** | **95% Confidence Interval** | | **P-value** | **n** |
| **History of CVD** | **Volume** | 1.93 | 1.52 | 2.45 | 7.45 x 10^-8^ | 881 | 1.65 | 1.20 | 2.27 | 0.002 | 526 |
|  | **Density** | 1.29 | 1.10 | 1.51 | 0.002 | 881 | 1.31 | 0.96 | 1.79 | 0.088 | 526 |
| **History of MI** | **Volume** | 3.13 | 2.14 | 4.59 | 4.63 x 10^-9^ | 881 | 3.46 | 1.84 | 6.52 | 1.18 x 10^-4^ | 547 |
|  | **Density** | 1.23 | 1.00 | 1.52 | 0.046 | 881 | 1.25 | 0.88 | 1.78 | 0.217 | 547 |

Table S5: Associations with incident all-cause and cardiovascular disease (CVD) mortality and history of CVD and myocardial infarction (MI) for an alternate coronary artery calcification density measure (Agatston over area) analyzed in independent models in European American and African American participants with type 2 diabetes. Hazard ratios for mortality associations and odds ratios for CVD and MI history reported per standard deviation change in coronary artery calcification measures. Models adjusted for age, sex, statin use, total cholesterol, HDL, systolic blood pressure, high blood pressure medication use, and current smoking; analyses additionally adjusted for study (Diabetes Heart Study or African American Diabetes Heart Study) in African Americans. All calcification measures used to calculate density were derived using a 90 HU (Hounsfield unit) threshold.

|  |  | **European Americans** | | | | | **African Americans** | | | | |
| --- | --- | --- | --- | --- | --- | --- | --- | --- | --- | --- | --- |
| **Outcome** | **CAC Measure** | **Hazard Ratio/ Odds Ratio** | **95% Confidence Interval** | | **P-value** | **n** | **Hazard Ratio/ Odds Ratio** | **95% Confidence Interval** | | **P-value** | **n** |
| **All-cause Mortality** | **Alternate Density Measure (Agatston over area)** | 1.36 | 1.19 | 1.56 | 9.62 x 10^-6^ | 901 | 1.23 | 0.97 | 1.56 | 0.092 | 551 |
| **CVD Mortality** | **Alternate Density Measure (Agatston over area)** | 1.32 | 1.10 | 1.58 | 0.003 | 886 | 1.55 | 1.09 | 2.20 | 0.014 | 543 |
| **History of CVD** | **Alternate Density Measure (Agatston over area)** | 1.79 | 1.52 | 2.13 | 1.05 x 10^-11^ | 893 | 1.74 | 1.40 | 2.16 | 4.66 x 10^-7^ | 525 |
| **History of MI** | **Alternate Density Measure (Agatston over area)** | 2.06 | 1.64 | 2.59 | 5.30 x 10^-10^ | 893 | 2.49 | 1.72 | 3.59 | 1.17 x 10^-6^ | 546 |

Table S6: Associations with incident all-cause and cardiovascular disease (CVD) mortality and history of CVD and myocardial infarction (MI) for an alternate coronary artery calcification density measure (Agatston over area) and volume measure analyzed in the same model in European American and African American participants with type 2 diabetes. Hazard ratios for mortality associations and odds ratios for CVD and MI history reported per standard deviation change in coronary artery calcification measures. Models adjusted for age, sex, statin use, total cholesterol, HDL, systolic blood pressure, high blood pressure medication use, and current smoking; analyses additionally adjusted for study (Diabetes Heart Study or African American Diabetes Heart Study) in African Americans. All calcification measures used to calculate density were derived using a 90 HU (Hounsfield unit) threshold.

|  |  | **European Americans** | | | | | **African Americans** | | | | |
| --- | --- | --- | --- | --- | --- | --- | --- | --- | --- | --- | --- |
| **Outcome** | **CAC measure** | **Hazard Ratio/ Odds Ratio** | **95% Confidence Interval** | | **P-value** | **n** | **Hazard Ratio/ Odds Ratio** | **95% Confidence Interval** | | **P-value** | **n** |
| **All-cause Mortality** | **Volume** | 1.60 | 1.21 | 2.11 | 0.001 | 901 | 1.61 | 1.00 | 2.62 | 0.052 | 551 |
|  | **Alternate Density Measure (Agatston over area)** | 1.04 | 0.85 | 1.28 | 0.686 | 901 | 0.88 | 0.57 | 1.36 | 0.577 | 551 |
| **CVD Mortality** | **Volume** | 2.24 | 1.51 | 3.33 | 6.86 x 10^-5^ | 886 | 2.04 | 0.83 | 5.03 | 0.120 | 543 |
|  | **Alternate Density Measure (Agatston over area)** | 0.84 | 0.63 | 1.11 | 0.223 | 886 | 0.96 | 0.46 | 2.00 | 0.922 | 543 |
| **History of CVD** | **Volume** | 1.92 | 1.42 | 2.59 | 2.14 x 10^-5^ | 893 | 1.70 | 1.17 | 2.46 | 0.006 | 525 |
|  | **Alternate Density Measure (Agatston over area)** | 1.17 | 0.92 | 1.48 | 0.196 | 893 | 1.20 | 0.86 | 1.67 | 0.287 | 525 |
| **History of MI** | **Volume** | 3.54 | 2.26 | 5.54 | 3.59 x 10^-8^ | 893 | 3.69 | 1.87 | 7.26 | 1.63 x 10^-4^ | 546 |
|  | **Alternate Density Measure (Agatston over area)** | 1.01 | 0.76 | 1.36 | 0.939 | 893 | 1.13 | 0.69 | 1.84 | 0.633 | 546 |

Table S7: Associations with incident all-cause and cardiovascular disease (CVD) mortality and history of CVD and myocardial infarction (MI) for coronary artery calcification measures analyzed in independent models in European American and African American participants with type 2 diabetes, with the analysis limited to those with a non-zero Agatston score at the 130 Hounsfield units (HU) threshold. Hazard ratios for mortality associations and odds ratios for CVD and MI history reported per standard deviation change in coronary artery calcification measures. Models adjusted for age, sex, statin use, total cholesterol, HDL, systolic blood pressure, high blood pressure medication use, and current smoking; analyses additionally adjusted for study (Diabetes Heart Study or African American Diabetes Heart Study) in African Americans. All calcification measures were derived using a 90 HU (Hounsfield unit) threshold.

|  |  | **European Americans** | | | | | **African Americans** | | | | |
| --- | --- | --- | --- | --- | --- | --- | --- | --- | --- | --- | --- |
| **Outcome** | **CAC Measure** | **Hazard Ratio/ Odds Ratio** | **95% Confidence Interval** | | **P-value** | **n** | **Hazard Ratio/ Odds Ratio** | **95% Confidence Interval** | | **P-value** | **n** |
| **All-cause Mortality** | **Volume** | 1.54 | 1.29 | 1.84 | 1.38 x 10^-6^ | 816 | 1.30 | 0.99 | 1.71 | 0.060 | 446 |
| **All-cause Mortality** | **Agatston score** | 1.54 | 1.29 | 1.85 | 3.36 x 10^-6^ | 816 | 1.28 | 0.98 | 1.67 | 0.067 | 446 |
| **All-cause Mortality** | **Density** | 1.28 | 1.10 | 1.49 | 0.002 | 812 | 1.00 | 0.80 | 1.26 | 0.991 | 446 |
| **CVD Mortality** | **Volume** | 1.75 | 1.35 | 2.28 | 3.15 x 10^-5^ | 801 | 1.94 | 1.24 | 3.03 | 0.004 | 438 |
| **CVD Mortality** | **Agatston score** | 1.71 | 1.30 | 2.25 | 1.43 x 10^-4^ | 801 | 1.91 | 1.22 | 3.00 | 0.005 | 438 |
| **CVD Mortality** | **Density** | 1.35 | 1.09 | 1.67 | 0.006 | 798 | 1.14 | 0.84 | 1.55 | 0.409 | 438 |
| **History of CVD** | **Volume** | 2.23 | 1.79 | 2.77 | 8.07 x 10^-13^ | 808 | 1.83 | 1.42 | 2.35 | 2.87 x 10^-6^ | 429 |
| **History of CVD** | **Agatston score** | 2.20 | 1.76 | 2.75 | 3.40 x 10^-12^ | 808 | 1.83 | 1.42 | 2.37 | 4.22 x 10^-6^ | 429 |
| **History of CVD** | **Density** | 1.63 | 1.37 | 1.93 | 3.59 x 10^-8^ | 804 | 1.82 | 1.36 | 2.44 | 5.58 x 10^-5^ | 429 |
| **History of MI*** | **Volume** | 3.35 | 2.46 | 4.58 | 2.53 x 10^-14^ | 809 | 3.34 | 1.96 | 5.71 | 9.87 x 10^-6^ | 443 |
| **History of MI*** | **Agatston score** | 3.36 | 2.39 | 4.71 | 2.38 x 10^-12^ | 809 | 3.46 | 1.91 | 6.27 | 4.13 x 10^-5^ | 443 |
| **History of MI** | **Density** | 1.71 | 1.34 | 2.19 | 2.24 x 10^-5^ | 805 | 2.10 | 1.52 | 2.89 | 5.90 x 10^-6^ | 443 |

*Model assumed independent, not exchangeable, correlation structure in African Americans.

Table S8: Associations with incident all-cause and cardiovascular disease (CVD) mortality and history of CVD and myocardial infarction (MI) for density and volume measures analyzed in the same model in European American and African American participants with type 2 diabetes, with the analysis limited to those with a non-zero Agatston score at the 130 Hounsfield unit (HU) threshold. Hazard ratios for mortality associations and odds ratios for CVD and MI history reported per standard deviation change in coronary artery calcification measures. Models adjusted for age, sex, statin use, total cholesterol, HDL, systolic blood pressure, high blood pressure medication use, and current smoking; analyses additionally adjusted for study (Diabetes Heart Study or African American Diabetes Heart Study) in African Americans. All calcification measures were derived using a 90 HU (Hounsfield unit) threshold.

|  |  | **European Americans** | | | | | **African Americans** | | | | |
| --- | --- | --- | --- | --- | --- | --- | --- | --- | --- | --- | --- |
| **Outcome** | **CAC measure** | **Hazard Ratio/ Odds Ratio** | **95% Confidence Interval** | | **P-value** | **n** | **Hazard Ratio/ Odds Ratio** | **95% Confidence Interval** | | **P-value** | **n** |
| **All-cause Mortality** | **Volume** | 1.51 | 1.24 | 1.83 | 4.00 x 10^-5^ | 812 | 1.51 | 1.05 | 2.15 | 0.025 | 446 |
|  | **Density** | 1.10 | 0.94 | 1.28 | 0.235 | 812 | 0.75 | 0.54 | 1.05 | 0.097 | 446 |
| **CVD Mortality** | **Volume** | 1.64 | 1.23 | 2.19 | 0.001 | 798 | 2.44 | 1.43 | 4.16 | 0.001 | 438 |
|  | **Density** | 1.13 | 0.92 | 1.40 | 0.248 | 798 | 0.65 | 0.40 | 1.06 | 0.082 | 438 |
| **History of CVD** | **Volume** | 2.06 | 1.63 | 2.62 | 1.86 x 10^-9^ | 804 | 1.56 | 1.16 | 2.10 | 0.003 | 429 |
|  | **Density** | 1.26 | 1.07 | 1.48 | 0.005 | 804 | 1.36 | 0.99 | 1.88 | 0.058 | 429 |
| **History of MI*** | **Volume** | 3.04 | 2.19 | 4.22 | 2.93 x 10^-11^ | 805 | 2.95 | 1.67 | 5.21 | 1.92 x 10^-4^ | 443 |
|  | **Density** | 1.22 | 0.99 | 1.51 | 0.065 | 805 | 1.28 | 0.89 | 1.83 | 0.180 | 443 |

*Model assumed independent, not exchangeable, correlation structure in African Americans.
